# The complete chloroplast genome and phylogenetic analysis of *Saussurea wettsteiniana* (Compositae)

**DOI:** 10.1080/23802359.2021.1939178

**Published:** 2021-06-14

**Authors:** Deng-Li Luo, Ying-Min Zhang, Ti-Cao Zhang, Guo-Dong Li

**Affiliations:** a Yunnan Key Laboratory for Dai and Yi Medicines, Yunnan University of Chinese Medicine, Kunming, People's Republic of China; b Faculty of Traditional Chinese Pharmacy, Yunnan University of Chinese Medicine, Kunming, People's Republic of China

**Keywords:** *Saussurea wettsteiniana*, chloroplast genome, medicinal plant, phylogenomics analysis

## Abstract

*Saussurea wettsteiniana* is a medicinally important herb endemic to Hengduan Mountains. Here, we report and characterize the complete chloroplast genome sequence of *S. wettsteiniana* to provide genomic resources useful for future study. The complete chloroplast genome is 152,631 bp in length, consisting of a large single copy and a small single copy of 83,552 bp and 18,637 bp, which were separated by a pair of inverted repeats of 25,221 bp. Totally 133 genes were annotated, including 87 protein-coding genes, 36 tRNA genes, and eight rRNA genes. We also detected two pseudo-genes (*ycf1* and *rps19*). The overall GC content of the whole genome is 37.7%. The phylogenetic tree based on 23 complete plastomes indicated that *S. wettsteiniana* was closely related to *S. involucrata* of Compositae.

*Saussurea wettsteiniana* Hand. - Mazz. is a perennial herb belonging to the Compositae family, endemic to Hengduan Mountains, and it is distributed in the elevation of 3400–4450 m of the hillside forests, forest edge, wet grassland and meadows (Flora of China Editorial Committee of Chinese Academy of Sciences 1999). It has been used in Tibetan folk medicine for the tonifying kidney, promoting blood circulation and strengthen Yang with a long history (Chen 2018). However, because of its low natural reproduction rate, overexploitation, and it is hard to cultivate artificially, natural populations of this species have suffered rapid declines. Besides, there is an extreme lack of research on the phylogenetic and conservation genetics of *S. wettsteiniana*. Here, we report and characterize the complete chloroplast genome sequence of *S. wettsteiniana.* It will provide reference data for the research on the phylogenetics and conservation genetics of *S. wettsteiniana*.

The fresh leaves of *S. wettsteiniana* were collected from Shangri-La County (27°52’N, 99°36’E), Yunnan province of China. A specimen was deposited at the Herbarium of Yunnan University of Chinese Medicine (YUNCM) (Guo-Dong Li, gammar116@163.com; Cong-Wei Yang, congweiyang@foxmail.com) under the voucher number 5334211256. The total genomic DNA was extracted using Plant Genomic DNA Extraction Kit (Bioteke Corporation, China) and sequencing was performed on the Illumina HiSeq 2500 platform (Illumina Inc., SanDiego, CA). All raw reads were filtered by using NGS QC Toolkit_v2.3.3 with default parameters to obtain clean reads (Patel and Jain 2012). Approximately 3.1 GB clean data were obtained and *de novo* assembled using NOVOPlasty 2.7.2 with complete genome of its close relative *Saussurea chabyoungsanica’s rbcL* gene (NC_036677) as the seed sequence (Dierckxsens et al. 2017). Gene annotation was performed with the online annotation tool GeSeq (Tillich et al. 2017) and subsequently corrected using Geneious Prime® 2020.1.1 The cpDNA sequence with complete annotation information was deposited at GenBank database under the accession number MW110638.

The complete chloroplast genome of *S. wettsteiniana* was a quadripartite circular and 152,631 bp in length, comprising a large single copy (LSC) region of 83,552 bp and a small single copy (SSC) region of 18,637 bp, separated by two inverted repeat (IR) regions of 25,221 bp. The chloroplast genome contained 133 genes, including 87 protein-coding genes, 36 tRNA genes, and eight rRNA genes. We also detected two pseudo-genes (*ycf1* and *rps19*). The overall GC-content of the whole cp genome is 37.7%, and those in the LSC, SSC, and IR regions are 35.8%, 38.6%, and 43.1%, respectively.

To identify the phylogenetic positions of *S. wettsteiniana*, as well as Compositae, we inferred the phylogenetic relationships based on the complete chloroplast genomes of 21 species from whole range of the family Asteraceae and two Codonopsis species as outgroups. The alignment was carried out by software MAFFT v.7 (Katoh and Standley 2013). The maximum-likelihood (ML) analyses were constructed using IQ-TREE v1.6.10 (Nguyen et al. 2015) with the best-fit model selected by Model Finder and 1000 bootstrap replicates for each branch node (Figure 1). The phylogenetic tree topology from the ML analysis showed that *S. wettsteiniana* and *S. involucrata* from Compositae formed a monophyletic clade with 100% bootstrap value. The complete chloroplast genome of *S.wettsteiniana* will provide a useful resource for the conservation genetics of this species as well as for the phylogenetic studies of Compositae.

**Figure 1.**
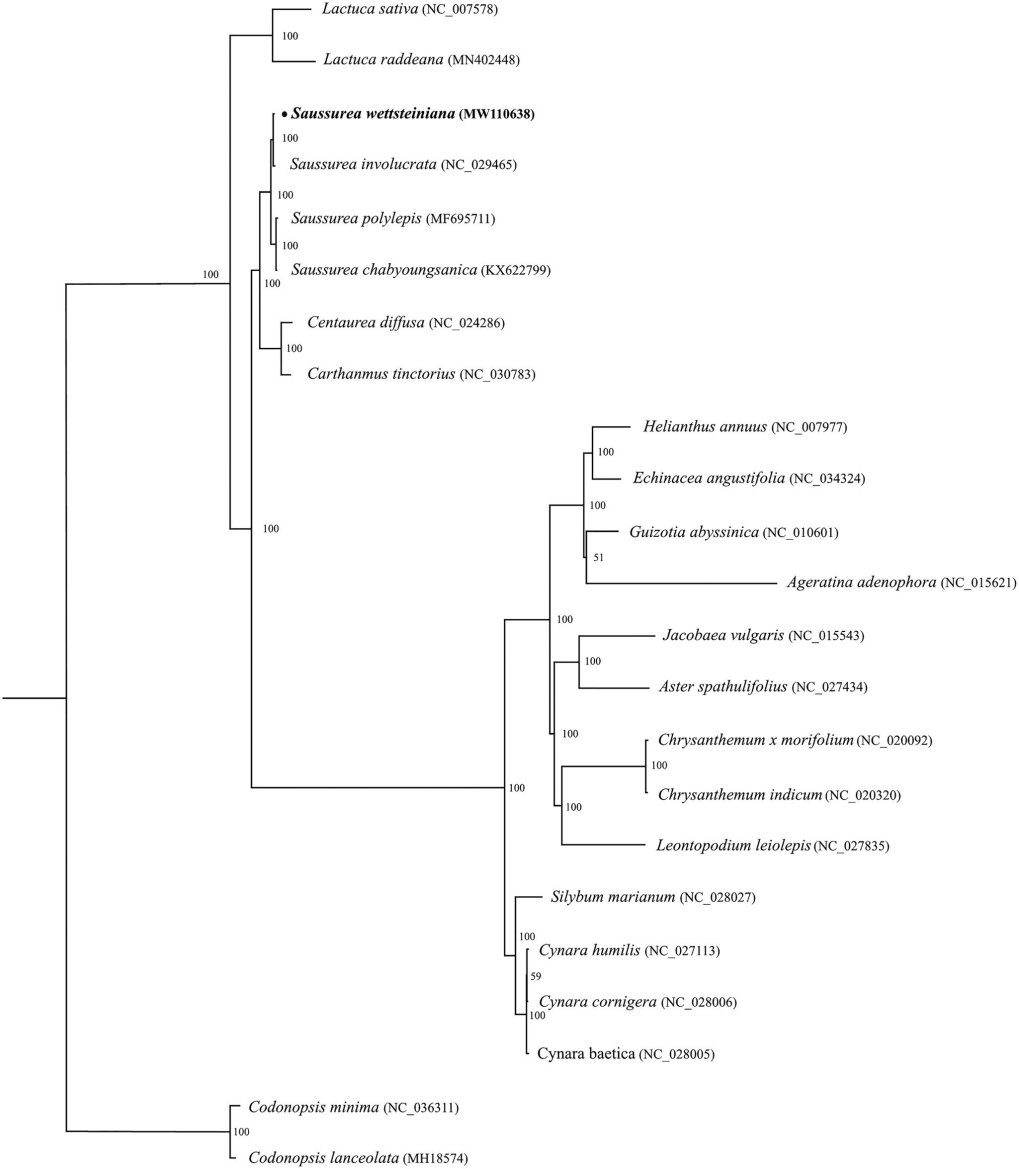
Maximum likelihood phylogenetic tree inferred from 23 chloroplast genomes. Bootstrap support values >50% are indicated next to the branches.

## Data Availability

The *Saussurea wettsteiniana* data that support the findings of this study are openly available in GenBank of NCBI at [https://www.ncbi.nlm.nih.gov] (https://www.ncbi.nlm.nih.gov/) under the accession no. MW110638. The associated BioProject, SRA, and Bio-Sample numbers are PRJNA721598, SUB9469822, and SAMN18721751, respectively.
